# TNF-α-308 G/A Polymorphism in Egyptian Budd-Chiari Syndrome Patients

**DOI:** 10.5505/tjh.2012.71473

**Published:** 2012-12-05

**Authors:** Yonca Eğin, Solaf Elsayed, Mohamed Sakr, Nejat Akar

**Affiliations:** 1 Ankara University, Department of Pediatric Genetics, Ankara, Turkey; 2 Ain Shams University, Pediatrics Department, Genetics Unit, Cairo, Egypt; 3 Ain Shams University, Cairo, Egypt; 4 TOBB Economy and Technology University Hospital, Ankara, Turkey

**To the Editor, **

Budd-Chiari syndrome (BCS) is an uncommon condition induced by thrombotic or non-thrombotic obstruction of hepatic venous outflow. BCS most often occurs in patients with underlying thrombotic diathesis, including such myeloproliferative disorders (MPDs) as polycythemia vera and paroxysmal nocturnal hemoglobinuria, and pregnancy, oral contraceptives, tumors, chronic inflammatory diseases, clotting disorders, and infections [1]. 

Tumor necrosis factor-alpha (TNF-α) is a pleiotropic cytokine produced primarily by macrophages and T-cells that has a range of inflammatory and immunomodulatory activity [2]. TNF-α is a pro-inflammatory cytokine with -308 promoter region G/A polymorphism. This polymorphism has been shown to affect the level of expression of the gene in vitro. Population-based studies on the effect of TNF-α -308 G/A on the occurrence of thromboembolic disease have reported inconsistent findings [3,4,5]. 

Recently, Fleischman et al. reported that Jak-2 V617F activity is positively correlated with TNF-α mRNA expression, suggesting that Jak2 V617F directly up regulates TNF-α mRNA in myeloproliferative neoplasm (MPN) patients [4]. As such, we aimed to study a polymorphic site located in the promotor region of the TNF-α gene (TNF- α -308 G/A) in Egyptian BCS patients and compared to an Egyptian control group. 

DNA was obtained from peripheral blood samples of BCS patients (n = 84) and healthy controls (n = 101). DNA was isolated using a MagnaPure LC 2.0 automatic isolation system (Roche Diagnostics, Rotkreuz, Switzerland). The TNF-α -308 promoter region was then amplified using specific primers. A 194-bp product was amplified using the primers, F: 5’-ATTGGAAATAGGTTTTGAGGGTCAT- 3’ and R:5’TCTCGGTTTCTTCTCCATCGC-3’ (MWG, Germany). These PCR products were digested using PagI (Fermantas, Lithuania) restriction enzyme. Re-stricted fragments were run on 3% agarose gel and viewed under UV light [3]. 

Among the 84 BCS patients, TNF-α -308 G/A polymorphism was present in 8.3% (n = 7), whereas the frequency of TNF-α -308 G/A polymorphism in the healthy Egyptian controls was 7.9%. The distribution of TNF-α -308 G/A polymorphisms in the BCS patients and controls is shown in the Table. 

Fleischman et al. reported that TNF-α plays a central role in promoting clonal dominance of Jak2 V617Fexpressing cells in MPN. They showed that Jak2 V617F kinase regulates TNF-α expression in cell lines and primary MPN cells, and that TNF-α expression is correlated with Jak2 V617F allele burden [4]. Ghaffar et al. recently reported that factor V Leiden (FVL) was a major etiological factor associated with thrombosis in Egyptian BCS patients, as compared to the frequency of FVL in the general Egyptian population, [6,7]. Elevated TNF-α might be associated with an increase in the risk of thrombotic complications due to the effect of this cytokine on the endothelium. The frequency of TNF-α -308 G/A polymorphism did not differ between Egyptian BCS patients and healthy controls in the present study. 

**Conflict of Interest Statement**

None of the authors have any conflicts of interest, including specific financial interests, relationships, and/or affiliations, relevant to the subject matter or materials included.

## Figures and Tables

**Table 1:**
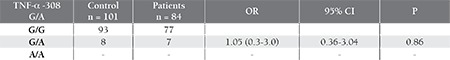
Distribution of TNF-α -308 G/A polymorphisms in the BCS patients and controls.

## References

[1] Shetty S, Ghosh K (2011). Thrombophilic dimension of Budd chiari syndrome and portal venous thrombosis-a concise review. Thromb Res.

[2] Allen RD (1999). Polymorphism of the human TNF-alpha promoter-random variation or functional diversity?. Mol Immunol.

[3] Akar N, Hasipek M (2002). Tumour Necrosis Factor-Alpha Gene Polymorphism (-308 G-A) in Turkish Pediatric Thrombosis Patients. Turk J Hematol.

[4] Fleischman AG, Aichberger KJ, Luty SB, Bumm TG, Petersen CL, Doratotaj S, Vasudevan KB, Latocha DH, Yang F, Press RD, Loriaux MM, Pahl HL, Silver RT, Agarwal A, O'Hare T, Druker BJ, Bagby GC, Deininger MW (2011). TNFα facilitates clonal expansion of JAK2V617F positive cells in myeloproliferative neoplasms. Blood.

[5] Elahi MM, Asotra K, Matata BM, Mastana SS (2009). Tumor necrosis factor alpha -308 gene locus promoter polymorphism: An analysis of association with health and disease. Biochim Biophys Acta.

[6] Abdel Ghaffar TY, Elsayed SM, Sakr MA, Elsobky ES, Egin Y, Akar N (2011). Factor V G1691A (Leiden) is a major etiological factor in Egyptian Budd- Chiari syndrome patients. Turk J Hematol.

[7] Ulu A, Elsobky E, Elsayed M, Yildiz Z, Tekin M, Akar N (2006). Frequency of five thrombophilic polymorphisms in the Egyptian population. Turk J Hematol.

